# B Lymphocytes in Latent Autoimmune Diabetes in Adults: From Autoimmune Activation to Immunometabolic Heterogeneity and Precision Therapeutic Perspectives

**DOI:** 10.1002/dmrr.70224

**Published:** 2026-08-27

**Authors:** Moncef Zouali

**Affiliations:** ^1^ Graduate Institute of Biomedical Sciences China Medical University Taichung Taiwan

**Keywords:** β‐cells, immune tolerance, molecular endotypes, precision medicine, regulatory B cells

## Abstract

Latent autoimmune diabetes in adults (LADA) is a heterogeneous form of autoimmune diabetes characterised by adult onset, the presence of islet autoantibodies, and a slower progression to insulin dependence compared with classical type 1 diabetes. Positioned at the interface of type 1 and type 2 diabetes, LADA arises from complex interactions among genetic susceptibility, immune dysregulation, and metabolic stress. While autoreactive T cells are recognized as central mediators of β‐cell destruction, accumulating evidence indicates that B lymphocytes play a broader and dynamic role in disease pathogenesis than previously appreciated. Beyond autoantibody production, B cells contribute to LADA through antigen presentation, cytokine secretion, and regulation of T cell responses, thereby amplifying autoimmune circuits within pancreatic islets. Emerging studies further demonstrate perturbations in circulating B cell subsets, including expansion of memory B cells and plasmablasts and impairment of regulatory B cells. However, whether these alterations represent primary drivers of disease progression or secondary responses to ongoing β‐cell injury remains unresolved. In parallel, immunometabolic stress and β‐cell dysfunction promote neoantigen formation and may shape B cell activation and selection, linking metabolic perturbations to adaptive immune dysregulation. Together, these findings support a model in which B lymphocytes act as both effector and regulatory components within a dynamic immunometabolic network that determines disease heterogeneity and progression. Recent advances in molecular profiling have further revealed distinct LADA endotypes, particularly stratified by glutamic acid decarboxylase antibody levels, suggesting divergent autoimmune and metabolic contributions across patient subgroups. These insights provide a rationale for precision medicine approaches and highlight B lymphocyte‐targeted interventions as potential, although still investigational, therapeutic strategies aimed at preserving residual β‐cell function.

## 1 Introduction

Latent autoimmune diabetes in adults (LADA) is a heterogeneous form of autoimmune diabetes characterised by adult‐onset disease, the presence of circulating islet autoantibodies, and progressive pancreatic β‐cell dysfunction ultimately leading to insulin dependence of circulating islet autoantibodies, and eventual progression to insulin dependence. Clinically, LADA occupies a position along the spectrum of autoimmune diabetes, sharing immunological characteristics with type 1 diabetes (T1D) while frequently exhibiting metabolic features commonly associated with type 2 diabetes (T2D), including insulin resistance and excess adiposity. Epidemiological studies estimate that LADA accounts for approximately 5–12% of adult diabetes cases, although the reported prevalence varies according to ethnicity, genetic background, and the extent of islet autoantibody screening performed in clinical practice [1]. Owing to its initial clinical resemblance to T2D, LADA remains frequently underrecognized and consequently undertreated during the early stages of the disease.

Current diagnostic criteria generally include adult‐onset diabetes, the presence of at least one islet autoantibody, and the absence of insulin requirement for several months after diagnosis [2]. However, these clinical criteria define a syndrome rather than a biologically homogeneous disease (Table 1). Increasing evidence indicates that LADA encompasses multiple immunological and metabolic phenotypes that differ in genetic susceptibility, degree of autoimmunity, β‐cell functional reserve, insulin resistance, and rate of progression towards insulin dependence [3, 4, 5]. This heterogeneity has stimulated growing interest in identifying molecular endotypes that may improve disease classification and facilitate precision therapeutic strategies.

**TABLE 1:** Overlapping characteristics of type 1 diabetes, latent autoimmune diabetes in adults, and type 2 diabetes.

	Type 1 diabetes	Latent autoimmune diabetes in adults	Type 2 diabetes
Family history	Variable	Variable	Frequent
Age of diagnosis	Generally childhood	Over 30–35	Variable
Body mass index	Generally normal	Generally normal	Over 25
HLA predisposing genes	Frequent	Frequent	Infrequent
β‐cell function	Lost	Diminished	Generally normal
Serum autoantibodies	Present	Present	Generally absent
Insulin requirement	Generally present	Generally present	Generally absent
Insulin resistance	Common	Delayed	Generally high

Historically, investigations of autoimmune diabetes have focused predominantly on T lymphocytes, particularly autoreactive CD8^+^ cytotoxic T cells and CD4^+^ helper T‐cell subsets responsible for β‐cell destruction [6]. Although these mechanisms remain fundamental to disease pathogenesis, they do not fully explain the considerable clinical heterogeneity observed in adult‐onset autoimmune diabetes, including differences in autoantibody profiles, progression rates, and the coexistence of metabolic dysfunction. These limitations have prompted increasing attention towards additional immune populations capable of integrating adaptive immune responses with metabolic stress.

Among these, B lymphocytes have emerged as important modulators of autoimmune diabetes. Beyond their classical role in antibody production, B cells function as highly efficient antigen‐presenting cells, cytokine‐producing immune effectors, and regulators of peripheral immune tolerance [7]. Most mechanistic insights into these functions have been derived from studies of classical T1D and experimental autoimmune diabetes models. Nevertheless, accumulating evidence suggests that similar immunological mechanisms may also contribute to disease progression in LADA, although direct mechanistic studies remain comparatively limited [7, 8]. Consequently, the precise contribution of B lymphocytes to adult‐onset autoimmune diabetes remains incompletely defined.

Recent studies have reported alterations in the peripheral B‐cell compartment of individuals with LADA, including expansion of memory B cells and plasmablasts together with reduced frequencies of naïve and regulatory B‐cell subsets. These changes have been associated with islet autoantibody profiles, declining β‐cell function, and progression towards insulin dependence. However, the available evidence is derived predominantly from cross‐sectional studies involving relatively small patient cohorts, and it remains uncertain whether these abnormalities represent primary drivers of disease or secondary adaptations to ongoing β‐cell injury.

Concurrently, advances in β‐cell biology have highlighted the importance of endoplasmic reticulum (ER) stress, inflammatory signalling, and metabolic overload in shaping autoimmune responses. Much of the mechanistic evidence regarding ER stress, neoantigen generation, and altered antigen presentation originates from studies in T1D; however, these pathways provide a biologically plausible framework for understanding how metabolic stress may amplify autoimmunity in LADA [9]. Rather than functioning solely as passive targets of immune attack, stressed β‐cells may actively contribute to immune activation by modifying antigen processing and promoting inflammatory signalling.

Taken together, these observations support an integrated immunometabolic model in which genetic susceptibility, β‐cell stress, metabolic dysfunction, and adaptive immune responses interact to determine disease progression. Within this framework, B lymphocytes may serve as important intermediaries linking metabolic stress with sustained autoimmune activation. This review critically evaluates current evidence regarding the role of B lymphocytes in LADA, distinguishing observations derived directly from LADA cohorts from mechanistic insights extrapolated from T1D and broader autoimmune diabetes research. Particular emphasis is placed on B‐cell‐mediated antigen presentation, alterations in B‐cell subsets, immunometabolic crosstalk, molecular heterogeneity, and emerging therapeutic strategies, while highlighting the major knowledge gaps that continue to limit the development of precision immunotherapies for adult‐onset autoimmune diabetes.

## 2 Pathogenic Mechanisms and B Lymphocyte Functions in LADA

**FIGURE 1:**
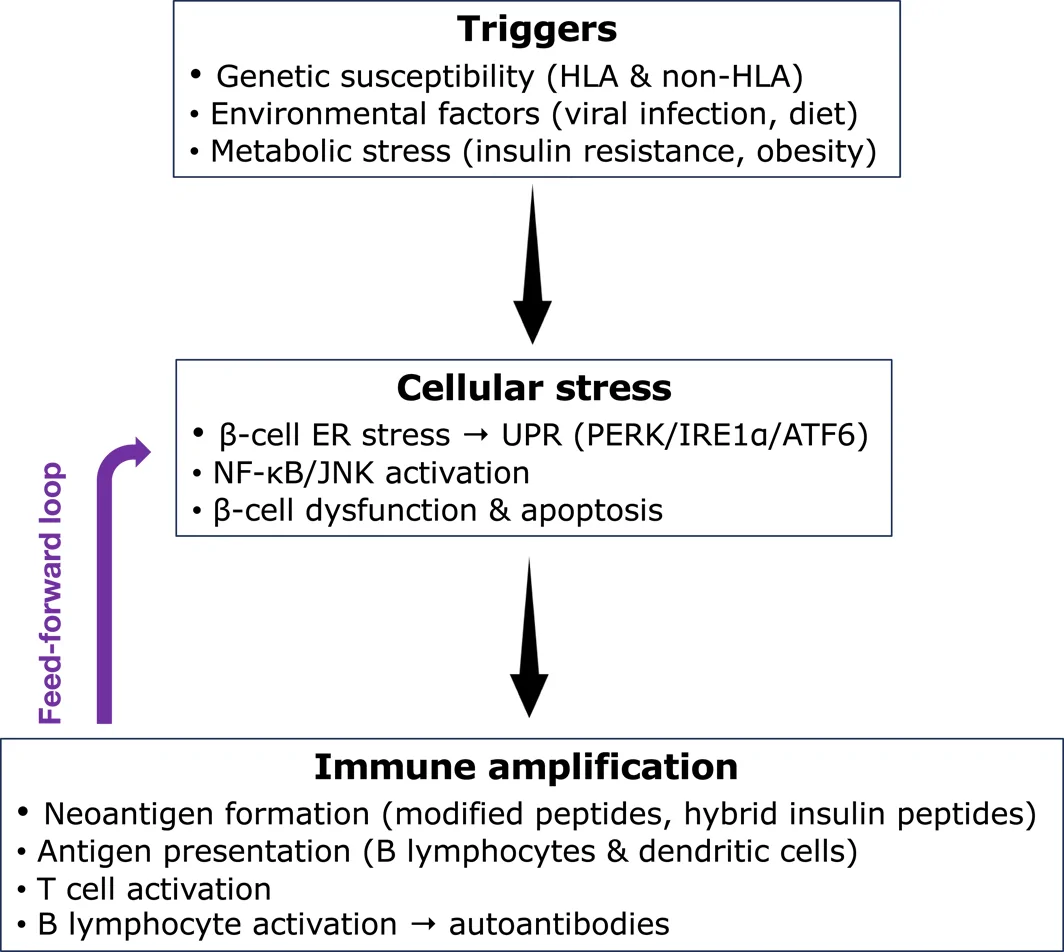
Immunometabolic circuit underlying LADA pathogenesis. LADA arises from a bidirectional interaction between genetic susceptibility, β‐cell stress, and immune dysregulation. Metabolic stress and inflammatory signalling induce endoplasmic reticulum dysfunction in pancreatic β‐cells, promoting neoantigen formation and enhanced antigen presentation. These stress‐induced changes facilitate activation of autoreactive B and T lymphocytes, initiating and sustaining a feed‐forward immune loop. Progressive immune‐mediated β‐cell injury further amplifies antigen release, reinforcing chronic inflammation and accelerating disease progression. B lymphocytes function as central immunological amplifiers in this process. They integrate β‐cell–derived antigenic signals and contribute to disease progression through antigen presentation to CD4^+^ T cells, autoantibody production, and cytokine secretion. These processes support the activation and maintenance of adaptive immune responses rather than serving as primary initiators of disease.

Latent autoimmune diabetes in adults develops through complex interactions among genetic susceptibility, environmental exposures, adaptive immune dysregulation, and metabolic stress that ultimately result in progressive pancreatic β‐cell loss (Figure 1). Although LADA shares many immunological features with classical T1D, including islet autoimmunity and HLA‐associated genetic susceptibility, its clinical course is typically more indolent and frequently accompanied by varying degrees of insulin resistance and other metabolic abnormalities [10]. Rather than representing a simple intermediate phenotype between T1D and T2D, LADA is increasingly regarded as a heterogeneous immunometabolic disorder in which the relative contributions of autoimmunity and metabolic dysfunction vary considerably among individuals.

Current understanding of autoimmune β‐cell destruction has been derived predominantly from studies of T1D and experimental models, where autoreactive T lymphocytes are recognized as the principal mediators of β‐cell injury. CD8^+^ cytotoxic T cells recognise β‐cell‐derived peptides presented by major histocompatibility complex (MHC) class I molecules and induce apoptosis of pancreatic β‐cells through perforin‐ and granzyme‐dependent mechanisms [11, 12]. CD4^+^ helper T cells further amplify islet inflammation by providing co‐stimulatory signals, producing pro‐inflammatory cytokines, and orchestrating interactions among antigen‐presenting cells, B lymphocytes, and innate immune populations. Although comparable mechanistic studies are limited in LADA, available histological, genetic, and immunological evidence suggests that similar immune pathways operate during disease progression, albeit with slower kinetics and greater influence of concomitant metabolic stress.

Within this pathogenic network, B lymphocytes are increasingly recognized as important modulators rather than passive producers of islet autoantibodies. In autoimmune diabetes, B cells contribute to adaptive immune responses through several complementary functions, including antigen presentation, cytokine secretion, and regulation of immune tolerance [8, 13]. Much of the mechanistic evidence supporting these functions originates from T1D and experimental autoimmune diabetes, whereas studies specifically examining B‐cell biology in LADA remain comparatively limited. Consequently, the extent to which individual B‐cell‐mediated mechanisms contribute to disease initiation, progression, or maintenance in LADA has not yet been fully established.

Genetic susceptibility provides the immunological framework within which these pathogenic mechanisms develop. Similar to T1D, LADA is strongly associated with HLA class II haplotypes, particularly HLA‐DR3‐DQ2 and HLA‐DR4‐DQ8, which influence antigen presentation to CD4^+^ T cells and shape the activation threshold of autoreactive lymphocytes [5, 14]. However, important quantitative differences distinguish LADA from childhood‐onset T1D. High‐risk DR3/DR4 heterozygosity occurs less frequently, whereas protective alleles such as DQB1*06:02 are more often retained. These genetic differences are thought to reduce the overall intensity of autoimmune responses and may contribute to the slower rate of β‐cell decline characteristic of many patients with LADA. Consistent with this concept, genetic risk scores for LADA generally occupy an intermediate position between those observed in T1D and T2D, further supporting the view that LADA represents a heterogeneous form of autoimmune diabetes rather than a distinct categorical disease.

In addition to HLA‐associated susceptibility, several non‐HLA loci implicated in immune regulation—including PTPN22, CTLA4, IL2RA, and SH2B3—have also been associated with LADA [3, 15]. These genes influence lymphocyte activation, co‐stimulatory signalling, cytokine responsiveness, and maintenance of peripheral immune tolerance. Conversely, polymorphisms traditionally associated with T2D susceptibility, particularly TCF7L2, are enriched in subsets of individuals with LADA [16], highlighting the co‐existence of autoimmune and metabolic mechanisms. The combination of intermediate HLA‐associated autoimmune risk together with variable metabolic susceptibility likely contributes to the marked clinical heterogeneity observed across the LADA spectrum.

Environmental factors may further modulate disease development in genetically susceptible individuals. Viral infections, particularly enteroviruses, have long been implicated in autoimmune diabetes because they can infect pancreatic β‐cells, induce local inflammation, and promote release of intracellular autoantigens [17, 18]. Although direct evidence linking viral infection to LADA remains limited, similar mechanisms are biologically plausible and may contribute to the initiation or amplification of autoimmunity. Likewise, obesity, insulin resistance, and chronic metabolic stress increase insulin demand, thereby enhancing β‐cell workload and vulnerability to inflammatory injury [19]. Rather than acting independently, these metabolic perturbations are increasingly viewed as amplifiers of ongoing autoimmune responses through their effects on β‐cell stress, antigen availability, and inflammatory signalling.

## 3 Functional Positioning of B Lymphocytes in LADA

B lymphocytes occupy a unique position at the interface between humoral and cellular immunity, allowing them to influence autoimmune responses through mechanisms extending well beyond antibody production [20]. In autoimmune diabetes, B cells contribute to immune activation by capturing antigen through the B‐cell receptor (BCR), presenting processed peptides to CD4^+^ T lymphocytes, producing cytokines, and regulating immune tolerance [21]. Although these functions have been extensively characterised in T1D and experimental autoimmune diabetes, direct mechanistic studies in LADA remain comparatively limited. Consequently, current understanding of B‐cell functions in LADA is derived from a combination of observations in LADA cohorts and mechanistic insights extrapolated from broader autoimmune diabetes research.

**FIGURE 2:**
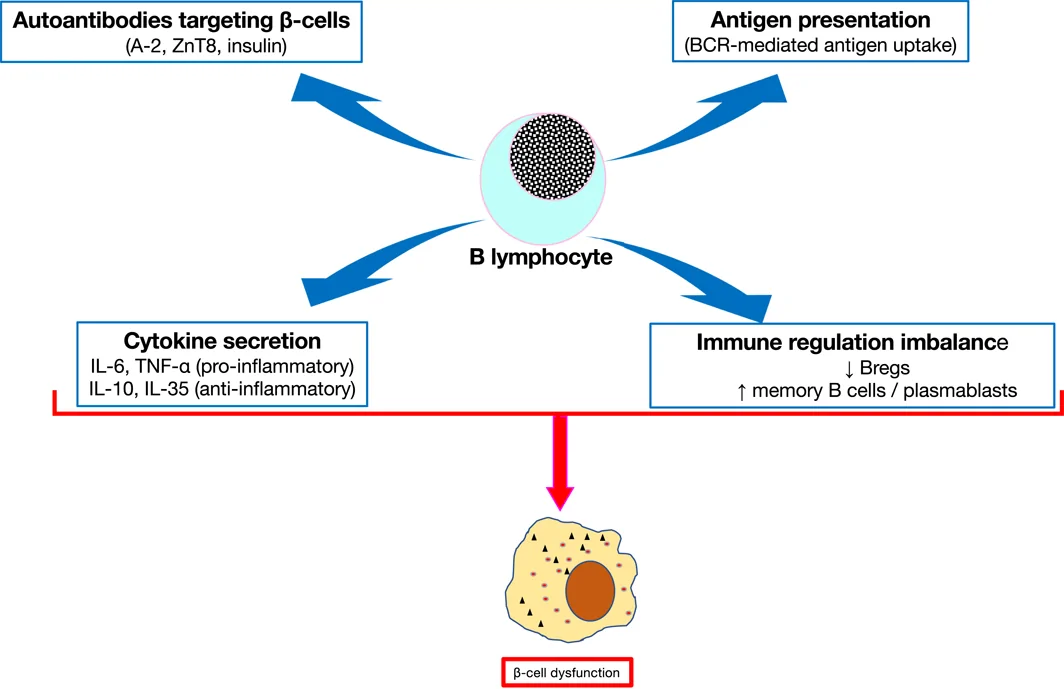
Multifaceted roles of B lymphocytes in LADA. Based on currently available evidence, B lymphocytes contribute to the pathogenesis of LADA through multiple interconnected functions that collectively amplify immune‐mediated β‐cell dysfunction. These include antigen presentation to CD4^+^ T cells via MHC class II, production of islet‐specific autoantibodies, and secretion of pro‐ and anti‐inflammatory cytokines. Alterations in B cell subsets, including expansion of memory B cells and plasmablasts and reduced regulatory B cell populations, are associated with heightened immune activation and reduced β‐cell function. Through these mechanisms, B lymphocytes integrate antigenic signals derived from stressed β‐cells and contribute to the maintenance and amplification of autoreactive T cell responses. This positions B lymphocytes as key immunological amplifiers within the immunometabolic network of LADA.

B lymphocytes originate from hematopoietic stem cells within the bone marrow, where sequential developmental checkpoints establish central immune tolerance by eliminating or functionally silencing highly autoreactive clones [22, 23, 24]. Additional peripheral tolerance mechanisms normally prevent activation of self‐reactive B cells that escape central deletion. Failure of these tolerance checkpoints has been implicated in multiple autoimmune diseases, including autoimmune diabetes, permitting survival and expansion of autoreactive B‐cell populations capable of recognising pancreatic β‐cell antigens. Whether defects in central or peripheral B‐cell tolerance are quantitatively or qualitatively different in LADA compared with classical T1D remains unknown and represents an important area for future investigation. Current evidence suggests that B lymphocytes may contribute to LADA through several complementary mechanisms (Figure 2), although the strength of supporting evidence differs substantially among these pathways.

## 4 Antigen Presentation

Beyond antibody production, B cells function as highly efficient antigen‐presenting cells. Following antigen recognition by the BCR, internalized proteins are processed and presented on MHC class II molecules to CD4^+^ T lymphocytes [25]. Compared with professional antigen‐presenting cells that rely on nonspecific antigen uptake, antigen‐specific B cells selectively concentrate cognate antigens through BCR‐mediated internalisation, substantially enhancing presentation efficiency even when antigen availability is limited. Most experimental evidence supporting this mechanism derives from T1D and animal models. Although similar pathways are likely to operate in LADA, direct functional studies demonstrating antigen presentation by autoreactive B cells in patients with LADA remain scarce. Thus, B‐cell‐mediated antigen presentation should currently be regarded as a biologically plausible contributor to disease progression rather than a mechanism definitively established in LADA.

## 5 Cytokine‐Mediated Immune Regulation

B lymphocytes also shape immune responses through cytokine secretion. Activated B cells produce pro‐inflammatory mediators including interleukin‐6 (IL‐6), tumour necrosis factor‐α (TNF‐α), and other cytokines capable of promoting T‐helper 1 (Th1) and T‐helper 17 (Th17) polarization, enhancing macrophage activation, and sustaining inflammatory immune responses [26, 27]. Conversely, regulatory B cells (Bregs) secrete anti‐inflammatory cytokines such as IL‐10 and IL‐35 that suppress excessive T‐cell activation and help maintain peripheral immune tolerance [28]. Most knowledge regarding these functional programmes derives from studies of T1D and other autoimmune disorders. In LADA, available evidence suggests similar alterations may occur, although the functional capacity of cytokine‐producing B‐cell subsets has been investigated in relatively few studies.

## 6 Functional Integration Within LADA

Rather than acting independently, these mechanisms are likely to operate in concert throughout disease progression. Autoantigen release resulting from β‐cell stress may promote activation of autoreactive B cells, which subsequently enhance T‐cell responses through antigen presentation while simultaneously shaping the inflammatory milieu through cytokine production. This integrated model provides a biologically plausible explanation for the close association between humoral autoimmunity, cellular immune activation, and progressive β‐cell dysfunction observed in LADA. Nevertheless, most available evidence remains associative, and longitudinal studies combining functional immune phenotyping with detailed clinical characterisation will be required to determine whether B‐cell abnormalities initiate disease, accelerate ongoing autoimmunity, or primarily reflect chronic immune activation.

## 7 B‐Cell Subset Dysregulation and Immune Imbalance in LADA

Advances in flow cytometry and immune phenotyping have revealed alterations in multiple peripheral B‐cell subsets in individuals with LADA. Although these findings support the concept that B‐cell homoeostasis is disturbed during disease progression, most available studies are cross‐sectional, involve relatively small patient cohorts, and employ different phenotypic definitions of B‐cell subsets. Consequently, current evidence is more consistent with an association between B‐cell dysregulation and disease progression than with a definitive causal role.

Several studies have reported reduced frequencies of naïve B cells together with expansion of antigen‐experienced memory B cells and plasmablasts in patients with LADA [29, 30]. Such changes are generally interpreted as reflecting chronic antigenic stimulation and sustained activation of autoreactive humoral immune responses. Increased plasmablast frequencies have been associated with higher islet autoantibody titres and lower fasting C‐peptide concentrations, suggesting a relationship between ongoing immune activation and progressive β‐cell dysfunction. However, comparable alterations have also been described in classical T1D and other autoimmune disorders, indicating that these changes are unlikely to be specific to LADA, but instead represent common features of chronic autoimmune activation.

Particular interest has focused on Bregs, a heterogeneous population that contributes to peripheral immune tolerance through the production of immunosuppressive cytokines, including interleukin‐10 (IL‐10), IL‐35, and transforming growth factor‐β (TGF‐β) [28]. Reduced frequencies and impaired suppressive function of Bregs have been reported in LADA and correlate with lower fasting C‐peptide concentrations and markers of disease progression [31, 32, 33]. Nevertheless, similar defects have also been documented in T1D and several systemic autoimmune diseases. Furthermore, Bregs comprise multiple phenotypically distinct populations—including transitional CD24hiCD38hi B cells, CD24hiCD27+ memory B cells, and IL‐10‐producing B10 cells—and no consensus currently exists regarding the most relevant regulatory subset in LADA. Standardisation of Breg phenotyping and functional assessment therefore remains an important priority for future studies.

Alterations in other B‐cell populations have also been described. Increased frequencies of marginal zone‐like B cells together with reductions in follicular B cells have been observed in some LADA cohorts when compared with individuals with T2D [29]. Because marginal zone B cells participate in early antigen recognition and bridge innate and adaptive immunity [34, 35], these findings have generated interest in their potential contribution to autoimmune activation. However, evidence remains limited, and whether these changes distinguish LADA from other forms of autoimmune diabetes or simply reflect ongoing immune activation has yet to be established.

Taken together, current evidence suggests that the overall pattern of B‐cell dysregulation in LADA closely resembles that observed in T1D, supporting the concept that these conditions share common mechanisms of autoimmune activation. The principal distinction appears to lie less in the nature of individual B‐cell abnormalities than in their clinical context. In LADA, B‐cell dysregulation develops within a more slowly evolving disease process that frequently coexists with insulin resistance, obesity, and chronic metabolic stress, creating an immunometabolic environment that may modify both the magnitude and tempo of autoimmune responses. Defining the features that are truly distinctive of LADA will require longitudinal studies integrating detailed immune phenotyping, functional analyses, and metabolic characterisation across the spectrum of adult‐onset autoimmune diabetes.

The next section shifts the focus from immune‐cell alterations to β‐cell biology. Rather than serving solely as passive targets of autoimmune attack, pancreatic β‐cells actively respond to metabolic and inflammatory stress. Emerging evidence—derived largely from studies of T1D but increasingly supported by observations in LADA—suggests that chronic endoplasmic reticulum stress, altered antigen processing, and neoantigen generation may enhance immune recognition and further amplify autoreactive B‐ and T‐cell responses. These mechanisms provide a biological framework linking metabolic dysfunction with progressive autoimmune activation.

## 8 Islet‐Specific Autoantibodies in LADA

Islet‐specific autoantibodies represent the most established biomarkers of autoimmune activity in LADA and provide indirect evidence of B‐cell involvement in disease pathogenesis. These antibodies arise following activation, differentiation, and maturation of autoreactive B cells into antibody‐secreting plasmablasts and plasma cells. Although autoantibodies are not generally considered primary mediators of β‐cell destruction, their presence reflects ongoing antigen recognition, epitope spreading, and maturation of humoral immune responses directed against pancreatic β‐cell antigens.

The most frequently detected autoantibody in LADA is glutamic acid decarboxylase autoantibody (GADA), which targets the GAD65 isoform expressed in pancreatic β‐cells [31, 32, 36]. Compared with other islet autoantibodies, GADA has the highest diagnostic sensitivity in adult‐onset autoimmune diabetes and often persists for many years after diagnosis. Additional autoantibody specificities include insulinoma‐associated antigen‐2 antibodies (IA–2A), zinc transporter‐8 antibodies (ZnT8A), insulin autoantibodies (IAA), and islet cell antibodies (ICA). The presence of multiple autoantibody specificities generally reflects broader epitope spreading and is associated with a higher probability of progressive β‐cell decline [37].

However, the biological significance of islet autoantibodies extends beyond simple positivity or negativity. Increasing evidence indicates that qualitative characteristics of the humoral immune response—including antigen specificity, epitope recognition patterns, antibody affinity, and maturation state—may provide additional information regarding disease heterogeneity. In this context, LADA is characterised by a predominantly GAD‐centred autoimmune response, but substantial variability exists among individuals in the magnitude and specificity of this response.

A landmark study demonstrated that differences in GAD65 antibody characteristics contribute to the clinical heterogeneity observed among adults with autoimmune diabetes [38]. Their findings suggested that qualitative differences in the autoimmune response against GAD65, including epitope recognition patterns, may distinguish individuals with more pronounced autoimmune phenotypes from those with slower disease progression. These observations support the concept that autoantibody profiles represent not only diagnostic markers but also indicators of underlying B‐cell activation pathways and immune maturation. Nevertheless, the extent to which specific antibody characteristics directly influence β‐cell decline remains uncertain.

GADA titre remains one of the most widely studied markers of disease heterogeneity. Higher GADA levels are generally associated with stronger autoimmune features, lower C‐peptide concentrations, increased enrichment of T1D‐associated HLA risk alleles, and faster progression towards insulin dependence [33, 37]. Conversely, individuals with lower GADA titres often exhibit greater overlap with T2D, including insulin resistance, obesity, and slower loss of β‐cell function. However, interpretation of GADA titres requires caution because thresholds vary among assays, including radiobinding assays and enzyme‐linked immunosorbent assays, limiting direct comparison between studies. Therefore, GADA concentration alone should not be considered a complete surrogate for autoimmune activity or future disease trajectory.

Beyond antibody quantity, antigenic specificity may provide additional insight into disease biology. Epitope mapping studies have identified preferential recognition of specific GAD65 domains in subsets of individuals with autoimmune diabetes, suggesting that antigen processing, B‐cell selection, and immune maturation shape the humoral response over time [39]. Similarly, the presence of multiple autoantibody specificities, including IA‐2A and ZnT8A, may indicate broader autoimmune activation and increased risk of β‐cell decline. However, the relationship between individual autoantibody characteristics and B‐cell functional states remains incompletely understood.

Collectively, islet autoantibodies provide an important window into B‐cell involvement in LADA but should be interpreted within a broader immunological and metabolic framework. Current evidence supports the concept that autoantibody profiles reflect the history and quality of autoimmune activation rather than acting as isolated determinants of disease progression. Future studies integrating antibody characteristics with B‐cell phenotyping, genetic risk, metabolic parameters, and longitudinal β‐cell function will be required to define clinically meaningful immune endotypes and improve patient stratification.

## 9 Immunometabolic Crosstalk and Neoantigen Formation in LADA

LADA develops within a complex immunometabolic environment in which autoimmune activation and metabolic stress interact to influence the rate of β‐cell decline. Unlike classical T1D, where immune‐mediated β‐cell destruction often dominates the clinical phenotype, many individuals with LADA exhibit concurrent insulin resistance, obesity, dyslipidemia, and features of metabolic syndrome. This coexistence suggests that β‐cell dysfunction in LADA may result from the combined effects of immune‐mediated injury and metabolic stress‐induced impairment of β‐cell resilience.

Pancreatic β‐cells are particularly susceptible to metabolic stress because of their high secretory demand and continuous requirement for insulin biosynthesis. Increased insulin requirements associated with insulin resistance can enhance endoplasmic reticulum (ER) workload, leading to accumulation of unfolded or misfolded proteins and activation of the unfolded protein response (UPR) [40]. Under physiological conditions, the UPR maintains cellular homoeostasis by reducing protein translation, increasing molecular chaperone activity, and promoting degradation of damaged proteins. However, persistent or excessive ER stress may shift this adaptive response towards cellular dysfunction, oxidative stress, inflammatory signalling, and apoptosis [41].

Most mechanistic evidence linking ER stress to autoimmune activation has been generated from T1D studies and experimental models. These investigations demonstrate that stressed β‐cells can exhibit altered antigen processing, increased expression of immune‐related molecules, and enhanced susceptibility to inflammatory injury. Although direct evidence in LADA remains limited, similar processes may be particularly relevant in this condition because metabolic stress and autoimmune susceptibility coexist within the same individuals. Thus, β‐cell stress may represent an important mechanism through which metabolic dysfunction modifies the tempo and severity of autoimmune progression.

Chronic β‐cell stress may also influence immune recognition by altering the antigenic landscape of pancreatic cells. Inflammatory and oxidative environments can promote post‐translational modifications of β‐cell proteins, including oxidation, deamidation, citrullination, and abnormal peptide processing [42]. These modifications may generate neoepitopes that differ from antigens encountered during immune tolerance development. In autoimmune diabetes, particular attention has been given to hybrid insulin peptides and modified proinsulin‐derived fragments, which can be recognized by autoreactive T cells and contribute to immune activation [12]. However, whether these neoantigen pathways are quantitatively or qualitatively different in LADA compared with T1D remains unresolved.

B lymphocytes may serve as important intermediaries linking β‐cell stress with adaptive immune activation. Through BCR‐mediated antigen recognition, autoreactive B cells can capture β‐cell‐derived antigens, process them, and present antigenic peptides to CD4^+^ T cells through MHC class II molecules. This antigen‐specific uptake allows B cells to efficiently amplify immune responses against low‐abundance autoantigens [43]. Although direct demonstration of neoantigen‐specific B‐cell responses in LADA is lacking, this mechanism provides a plausible explanation for how metabolic stress‐induced changes in β‐cell antigenicity could enhance autoreactive immune activation.

Metabolic inflammation occurring outside the pancreas may further amplify these processes. In obesity and insulin‐resistant states, adipose tissue undergoes inflammatory remodelling characterised by immune‐cell infiltration and altered secretion of adipokines and inflammatory mediators, including leptin, IL‐6, and TNF‐α [44]. These signals can promote inflammatory T‐cell polarization, enhance antigen‐presenting cell activation, and increase systemic oxidative stress. Elevated free fatty acids and reactive oxygen species may additionally worsen β‐cell ER stress and mitochondrial dysfunction. Therefore, metabolic dysfunction may not simply coexist with autoimmunity in LADA but may actively influence the inflammatory environment in which autoimmune responses evolve.

Emerging evidence also suggests that epigenetic mechanisms contribute to the immunometabolic interface in LADA. Altered DNA methylation patterns involving immune regulatory pathways have been reported in immune cells from individuals with LADA, including genes associated with cytokine signalling and immune activation [45]. In addition, microRNAs such as miR‐21 and miR‐146a have been implicated in inflammatory regulation, immune‐cell differentiation, and cellular stress responses. However, the extent to which these molecular changes represent causes of disease progression or consequences of chronic inflammation remains unclear.

Collectively, current evidence supports an integrated model in which genetic susceptibility, metabolic stress, β‐cell dysfunction, and immune activation interact to determine disease progression in LADA. Within this framework, stressed β‐cells may increase immune visibility through altered antigen processing and inflammatory signalling, while autoreactive B and T lymphocytes maintain chronic immune activation. Importantly, many mechanistic components of this model are supported primarily by T1D research, and their specific contribution to LADA requires further validation through longitudinal studies incorporating immune profiling, metabolic characterisation, and functional analyses. Nevertheless, the immunometabolic framework provides a valuable basis for understanding why LADA exhibits substantial heterogeneity and why combined immune and metabolic therapeutic strategies may be required.

## 10 Molecular Endotypes and Precision Immunology

**FIGURE 3:**
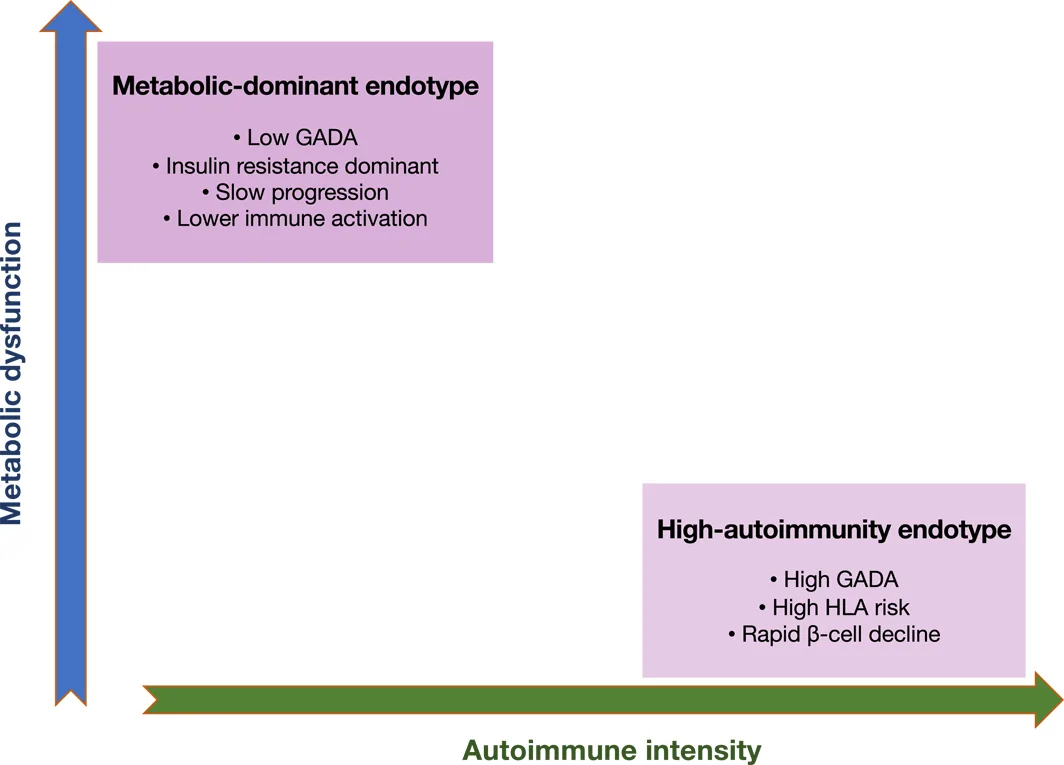
Immunometabolic endotypes of LADA along a GADA‐defined spectrum. LADA exhibits immunological and metabolic heterogeneity that can be conceptualised along a spectrum of glutamic acid decarboxylase antibody (GADA) titres, reflecting varying degrees of autoimmune and metabolic contributions rather than discrete disease subtypes. High‐GADA LADA is generally associated with stronger autoimmune activity, more pronounced B lymphocyte–mediated immune activation, and a more rapid decline in β‐cell function. This endotype is characterised by enhanced humoral immune responses and a greater contribution of immune‐mediated mechanisms. Low‐GADA LADA more frequently overlaps with metabolic features, such as insulin resistance, and displays comparatively weaker autoimmune signatures and slower progression of β‐cell dysfunction. These endotypes are not defined by absolute thresholds, but represent a continuum of immunometabolic activity shaped by interactions between β‐cell stress, immune activation, and metabolic dysregulation. B lymphocyte activity varies across this spectrum, contributing differentially to immune amplification and disease progression. The figure depicts a conceptual continuum and should not be interpreted as representing universally accepted GADA cutoffs.

Increasing evidence indicates that adult‐onset autoimmune diabetes represents a heterogeneous spectrum rather than a single biological entity. Although LADA is currently defined clinically by age at diagnosis, autoantibody positivity, and initial insulin independence, these criteria encompass individuals with substantial variation in genetic susceptibility, immune activation, metabolic phenotype, and rate of β‐cell decline. Therefore, molecular stratification approaches are increasingly being explored to identify clinically meaningful subgroups that may differ in disease trajectory and therapeutic response (Figure 3).

Transcriptomic and immune profiling studies have demonstrated substantial heterogeneity across autoimmune diabetes populations. Recent analyses suggest that individuals with LADA may exhibit intermediate inflammatory signatures between classical T1D and T2D, with differences observed in pathways related to cytokine signalling, immune activation, and cellular stress responses [46]. However, most available molecular datasets contain limited numbers of well‐characterised LADA cohorts, and many observations are derived from broader autoimmune diabetes populations rather than exclusively from LADA. Therefore, whether LADA represents a distinct molecular entity or an intermediate position within the broader spectrum of adult‐onset autoimmune diabetes remains an active area of investigation.

One commonly proposed approach to LADA stratification involves the characterisation of GAD autoantibody profiles. Individuals with higher GADA titres often display stronger autoimmune features, including lower C‐peptide levels, increased frequency of T1D‐associated genetic risk variants, and more rapid progression towards insulin dependence [33, 37]. Conversely, individuals with lower GADA titres frequently exhibit greater metabolic overlap with T2D, including increased insulin resistance, higher BMI, and slower decline in β‐cell function. These observations suggest that GADA levels may provide useful information regarding the relative contribution of autoimmune and metabolic mechanisms.

However, GADA titre alone should not be considered a definitive molecular endotype. Thresholds defining ‘high’ and ‘low’ GADA vary among studies and are influenced by assay methodology, including differences between radiobinding assays and ELISA‐based approaches. Furthermore, antibody quantity does not fully capture the complexity of B‐cell responses, including antigen specificity, affinity maturation, and immune‐cell interactions. Consequently, GADA profiles should be interpreted together with additional clinical and biological parameters rather than used as an isolated classification tool.

A more comprehensive precision medicine framework for LADA will likely require integration of multiple dimensions, including autoimmune markers (GADA titre, autoantibody multiplicity, epitope specificity, and other indicators of humoral immune activation); β‐cell functional measures (C‐peptide secretion, residual β‐cell reserve, and rate of decline over time); metabolic characteristics (BMI, insulin resistance, lipid abnormalities, and metabolic syndrome features); genetic factors (HLA‐associated autoimmune risk and polygenic susceptibility profiles); and immune phenotypes (B‐cell subset composition, regulatory immune‐cell function, and inflammatory signatures).

Such integrated approaches may allow identification of clinically relevant patient groups, including individuals with predominantly autoimmune‐driven disease who may benefit from immunomodulatory strategies and individuals with stronger metabolic contributions who may require approaches targeting insulin resistance and β‐cell stress.

## 11 Emerging Multi‐Omics Approaches for LADA

Recent advances in multi‐omics technologies have provided new opportunities to investigate the molecular complexity of autoimmune diabetes. Transcriptomics, epigenomics, chromatin accessibility profiling, proteomics, and metabolomics can collectively reveal regulatory networks linking immune activation, β‐cell dysfunction, and metabolic stress [47, 48]. In autoimmune diabetes research, single‐cell approaches have been particularly valuable for resolving heterogeneity among immune‐cell populations and identifying disease‐associated cellular states.

However, the application of multi‐omics specifically to LADA remains at an early stage. Many available datasets originate from T1D, T2D, or mixed adult autoimmune diabetes cohorts rather than carefully phenotyped LADA populations. These studies provide important mechanistic insights, but require cautious interpretation when applied to LADA. Future LADA‐focused investigations incorporating longitudinal sampling and integrated immune‐metabolic profiling will be essential to determine whether specific molecular signatures distinguish LADA from other forms of diabetes.

Single‐cell multi‐omics approaches may be particularly informative for understanding B‐cell involvement in LADA. These methods can characterise B‐cell developmental states, identify activated or regulatory populations, evaluate antigen‐receptor repertoires, and investigate interactions between B cells, T cells, and β‐cell stress pathways. Such approaches may help determine whether specific B‐cell states are associated with rapid β‐cell decline, persistent autoimmunity, or therapeutic responsiveness.

Ultimately, precision immunology in LADA will require moving beyond single biomarkers towards integrated biological models. The future goal is not simply to classify patients as having ‘high autoimmune’ or ‘low autoimmune’ disease, but to define mechanistically meaningful profiles that explain individual differences in disease progression and guide treatment selection. Achieving this objective will require large, longitudinal, deeply phenotyped LADA cohorts combining clinical data with immune, genetic, metabolic, and multi‐omics analyses.

## 12 Therapeutic Implications and Current Limitations

Recognition of B‐cell involvement in autoimmune diabetes has stimulated interest in therapies that modulate B‐cell activity to preserve residual β‐cell function (Table 2). However, therapeutic evidence directly generated in LADA remains extremely limited. Most available data come from clinical trials in recent‐onset T1D or experimental autoimmune diabetes models, where B cells have been implicated in antigen presentation, immune amplification, and regulation of autoreactive T‐cell responses. Although these findings provide a biological rationale for considering B‐cell‐directed approaches in LADA, differences in disease tempo, age at onset, metabolic background, and residual β‐cell function require cautious interpretation.

**TABLE 2:** B lymphocyte–targeted therapeutic strategies in autoimmune diabetes.

Mechanistic rationale	Therapeutic target/strategy	Limitations and considerations in LADA
Depletion of CD20^+^ B cells reduces antigen presentation, autoantibody production, and T cell co‐stimulation, thereby interrupting the autoimmune amplification loop	Anti‐CD20 monoclonal antibodies (e.g., rituximab, ocrelizumab)	Evidence primarily derived from T1D. Benefits appear transient with B cell repopulation. Potential risk of immunosuppression and limited data in LADA‐specific cohorts
Survival and maturation of autoreactive B cells is supported by BAFF/APRIL signalling, sustaining humoral autoimmunity	BAFF/APRIL pathway inhibitors (e.g., belimumab and investigational agents)	Clinical efficacy in diabetes remains unproven. Risk of broad B cell suppression affecting protective humoral immunity. Unclear impact on β‐cell preservation in LADA
CD40–CD40L interactions promote B cell activation, germinal centre formation, and T cell co‐stimulation, reinforcing adaptive immune responses	CD40/CD40L blockade (monoclonal antibodies or ligand inhibitors)	Safety concerns related to thromboembolic risk (notably with CD40L targeting). Limited clinical data outside other autoimmune diseases
Dysregulated BCR signalling lowers activation thresholds for autoreactive B cells, enabling sustained activation against β‐cell antigens	B Cell receptor signalling modulation (e.g., SYK, BTK inhibitors under investigation)	Potential off‐target effects on global B cell function. Limited clinical translation in autoimmune diabetes. Long‐term safety in metabolic disease unknown
Defective immune tolerance due to reduced regulatory B cell (Breg) number and function contributes to persistent inflammation	Enhancement or adoptive modulation of regulatory B cells (e.g., IL‐10–inducing strategies, cellular therapies)	Still experimental. Challenges in selective expansion of functional Bregs without enhancing pathogenic B cells. No established clinical protocols in LADA
Broad autoimmune activation is sustained by T cell–B cell interactions and cytokine networks	Combination immunomodulatory strategies targeting B–T cell interaction axes	Risk of over‐immunosuppression. Unclear optimal timing in disease course. Heterogeneity of LADA endotypes complicates patient stratification

## 13 B‐Cell Depletion and Immune Modulation

The most extensively studied B‐cell‐targeted strategy is depletion of CD20^+^ B cells using monoclonal antibodies such as rituximab. In recent‐onset T1D, anti‐CD20 therapy resulted in transient preservation of endogenous insulin secretion, as reflected by improved C‐peptide responses, supporting the concept that B cells contribute actively to autoimmune β‐cell injury [49]. In other studies, an anti‐CD22 calicheamicin‐conjugated monoclonal antibody that efficiently depletes mature B‐cells in mice was used to establish a therapeutic approach for T1D. In rodents, CD22 targeting depleted B‐cells and reversed experimental diabetes, thereby providing a blueprint for the development of novel therapies to cure autoimmune diabetes [50]. Mechanistically, B‐cell depletion may reduce antigen presentation to autoreactive T cells, decrease inflammatory cytokine production, and limit the expansion of activated memory B‐cell populations.

Nevertheless, extrapolation of these findings to LADA requires caution. Unlike early‐onset T1D, many individuals with LADA are diagnosed after substantial β‐cell decline has already occurred, potentially limiting the therapeutic window during which immune intervention can preserve meaningful β‐cell mass. In addition, rituximab primarily targets circulating CD20^+^ B cells and has limited effects on long‐lived plasma cells, which lack CD20 expression and may continue producing autoantibodies after treatment. The broader consequences of B‐cell depletion, including infection risk, reduced vaccine responses, and impaired protective immunity, also require consideration when evaluating long‐term treatment strategies.

Future studies will need to determine whether selected LADA subgroups—particularly individuals with strong autoimmune signatures, high‐risk HLA profiles, multiple autoantibodies, and preserved C‐peptide secretion—may derive greater benefit from B‐cell‐directed therapies. Such approaches are unlikely to be appropriate as universal treatment strategies for all individuals currently classified as having LADA.

## 14 Targeting B‐Cell Survival and Activation Pathways

Beyond direct depletion, modulation of B‐cell survival pathways represents another potential therapeutic approach. B‐cell activating factor (BAFF, also known as BLyS) and a proliferation‐inducing ligand (APRIL) regulate B‐cell maturation, survival, and plasma‐cell differentiation [51, 52]. Altered BAFF signalling has been implicated in several autoimmune disorders by promoting the survival of autoreactive B‐cell populations.

However, the relevance of BAFF/APRIL pathways in LADA remains uncertain. Because these pathways are also essential for maintaining normal humoral immunity, systemic inhibition may compromise immune protection. Identifying patients with dysregulated B‐cell survival pathways and developing biomarkers capable of predicting therapeutic response will be necessary before such approaches can be considered clinically applicable.

Similarly, targeting immune co‐stimulatory pathways such as CD40–CD40L interactions may theoretically reduce B‐cell/T‐cell collaboration and autoimmune amplification [53]. However, previous clinical experience with CD40L blockade has highlighted safety concerns, including thromboembolic complications, emphasising the need for selective rather than broad immune suppression.

## 15 Restoring Immune Tolerance Through Regulatory B Cells

An alternative therapeutic concept is the restoration of immune tolerance rather than elimination of immune cells. Regulatory B cells (Bregs) represent a heterogeneous group of immunoregulatory populations capable of suppressing inflammatory responses through secretion of IL‐10, IL‐35, and other regulatory mediators [54]. Reduced frequency or impaired function of Bregs has been reported in LADA and other autoimmune diabetes populations, suggesting that restoration of regulatory immune balance may represent a complementary therapeutic strategy.

However, several challenges remain before Breg‐based therapies can be translated clinically. Bregs are not defined by a single universally accepted phenotype, and populations described as regulatory include transitional CD24hiCD38hi B cells, CD24hiCD27+ memory B cells, and IL‐10‐producing B10 cells. It remains unclear which subset is most relevant to LADA pathogenesis, whether these populations can be selectively expanded, and how their stability can be maintained within a metabolically inflammatory environment. Future strategies may involve ex vivo expansion, adoptive transfer, or targeted manipulation of signalling pathways that promote regulatory function while minimising unwanted immunosuppression.

## 16 Integrating Metabolic and Immune Interventions

Because LADA develops through interaction between autoimmune activation and metabolic stress, therapies targeting immune pathways alone may not fully address disease progression. Persistent insulin resistance, obesity, glucotoxicity, lipotoxicity, and β‐cell stress may continue to promote dysfunction even when immune activation is partially controlled.

Therefore, therapies with immunometabolic effects may represent an important complementary strategy. In addition to improving glycaemic control, some glucose‐lowering therapies may influence inflammatory pathways, oxidative stress, and β‐cell stress responses. For example, a clinical study evaluating the GLP‐1 receptor agonist dulaglutide in individuals with LADA suggested potential benefits in preserving glycaemic control and influencing β‐cell function parameters, supporting the possibility that metabolic intervention may modify disease progression in selected patients [55]. However, whether such therapies directly influence B‐cell‐mediated autoimmunity remains unclear, and larger mechanistic studies are required.

This immunometabolic perspective suggests that future treatment strategies may require combination approaches. Patients with predominant autoimmune features may benefit from carefully selected immunomodulatory therapies, whereas individuals with greater metabolic dysfunction may require interventions focused on insulin sensitivity, β‐cell protection, and reduction of metabolic stress. Precision treatment selection will likely depend on integrating immune biomarkers, metabolic characteristics, genetic risk, and longitudinal measures of β‐cell function.

## 17 Current Limitations and Future Directions

Several limitations currently restrict the development of targeted therapies for LADA. First, the biological definition of LADA remains heterogeneous, and current diagnostic criteria identify a clinically diverse population rather than a uniform pathogenic entity. Second, many mechanistic insights regarding B‐cell biology originate from T1D studies, and their specific relevance to LADA requires validation. Third, reliable biomarkers capable of predicting disease progression, therapeutic responsiveness, or immune remission remain unavailable.

Future studies should prioritise longitudinally deeply phenotyped LADA cohorts incorporating immune profiling, single‐cell approaches, metabolomics, genetic analysis, and functional assays. Such studies will be essential to determine which B‐cell abnormalities represent drivers of disease progression, which reflect secondary immune responses, and which patient populations are most likely to benefit from targeted intervention.

Overall, B‐cell‐directed therapies represent promising but experimental approaches in LADA. The most effective therapeutic strategies will likely emerge not from broad immune suppression alone, but from individualised interventions that integrate B‐cell biology with metabolic regulation and preservation of β‐cell function.

## 18 Limitations of Current Evidence and Future Research Priorities

Despite increasing recognition of B‐cell involvement in LADA, several limitations currently restrict definitive conclusions regarding their pathogenic role and therapeutic relevance. First, most available studies investigating B‐cell function, antigen presentation, cytokine production, and immune regulation in autoimmune diabetes have been performed in classical T1D or experimental models rather than in well‐characterised LADA cohorts. Although these findings provide important mechanistic insights, the extent to which they directly apply to LADA remains incompletely established.

Second, current evidence describing B‐cell abnormalities in LADA is largely derived from cross‐sectional studies involving relatively small patient populations. Alterations in memory B cells, plasmablasts, and regulatory B cells may reflect active participation in disease progression, but they may also represent secondary responses to ongoing β‐cell injury or chronic inflammation. Longitudinal studies are therefore required to determine whether specific B‐cell signatures predict disease progression, β‐cell decline, or treatment response.

Third, the biological definition of LADA remains heterogeneous. Current diagnostic criteria identify a clinically defined population that includes individuals with varying degrees of autoimmune activity, genetic susceptibility, metabolic dysfunction, and residual β‐cell function. Consequently, studies combining diverse patient groups may obscure biologically meaningful subtypes. Future research should integrate immune phenotyping, autoantibody characteristics, genetic risk, metabolic parameters, and longitudinal C‐peptide measurements to establish clinically relevant molecular endotypes.

Finally, the translation of B‐cell biology into therapeutic applications remains challenging. Although B‐cell‐targeted therapies have demonstrated proof‐of‐concept in T1D, dedicated clinical trials in LADA are scarce, and optimal patient selection, treatment timing, and combination strategies remain undefined. Future investigations using single‐cell immune profiling, B‐cell receptor repertoire analysis, multi‐omics approaches, and functional immune assays will be essential to identify pathogenic B‐cell populations and develop precision interventions that balance immune modulation with preservation of protective immunity.

## 19 Conclusions

LADA represents a heterogeneous form of adult‐onset autoimmune diabetes in which genetic susceptibility, immune dysregulation, β‐cell stress, and metabolic dysfunction interact to determine disease progression. Rather than representing simply a slowly progressive form of classical T1D or a subtype of T2D, LADA appears to occupy a complex position within the broader spectrum of autoimmune and metabolic diabetes. This framework helps explain the substantial variation observed among individuals in autoimmune profiles, residual β‐cell function, metabolic characteristics, and rates of progression towards insulin dependence.

Within this interconnected network, B lymphocytes represent important regulators of disease‐associated immune responses. Beyond their traditional role as producers of islet autoantibodies, B cells can contribute to autoimmune diabetes through antigen presentation, cytokine production, interaction with autoreactive T cells, and regulation of immune tolerance. Studies in LADA have identified alterations in B‐cell subsets, including changes in memory B cells, plasmablasts, and regulatory B‐cell populations. However, many of these observations overlap with findings in classical T1D and other autoimmune diseases, and whether these abnormalities represent initiating mechanisms, disease amplifiers, or secondary consequences of β‐cell injury remains unresolved.

The relationship between B‐cell biology and β‐cell stress provides a potential framework for understanding the immunometabolic complexity of LADA. Metabolic stress, insulin resistance, and inflammatory signalling may alter β‐cell function and antigen presentation, thereby influencing immune recognition and sustaining autoreactive responses. Conversely, immune activation may accelerate β‐cell dysfunction and amplify metabolic disturbances. Within this bidirectional model, B cells may function as important intermediaries connecting metabolic stress with adaptive immune activation.

Current evidence also supports the existence of clinically meaningful heterogeneity within LADA. Autoantibody profiles, particularly GADA characteristics, provide valuable information regarding autoimmune activity, but are unlikely to define complete disease endotypes when considered alone. Future classification strategies will likely require integration of humoral immune characteristics, B‐cell phenotypes, genetic susceptibility, metabolic parameters, C‐peptide trajectories, and molecular signatures. Such approaches may enable a more precise prediction of disease progression and identification of individuals most likely to benefit from targeted therapies.

Although B‐cell‐directed interventions represent a promising therapeutic concept, their application in LADA remains investigational. Most evidence supporting B‐cell‐targeted therapy originates from T1D studies, and dedicated LADA clinical trials remain limited. Future therapeutic strategies will likely require individualised approaches that consider both immune mechanisms and metabolic contributors to β‐cell failure. Combination strategies integrating immune modulation, metabolic intervention, and β‐cell preservation may ultimately provide greater benefit than approaches targeting a single pathway.

Several important knowledge gaps remain. Large longitudinal LADA cohorts with comprehensive immune and metabolic characterisation are needed to determine the temporal relationship between B‐cell alterations, metabolic stress, and β‐cell decline. Improved definition of regulatory B‐cell populations, characterisation of autoreactive B‐cell repertoires, and application of single‐cell and multi‐omics technologies may clarify which immune pathways are most relevant for therapeutic targeting. Furthermore, the development of validated biomarkers will be essential for identifying disease endotypes and guiding precision treatment strategies.

In conclusion, B lymphocytes represent a significant component of the immunopathological landscape of LADA, but their role should be understood within the broader context of immune–metabolic interactions. A more integrated understanding of B‐cell biology, β‐cell stress responses, and metabolic dysfunction may ultimately shift LADA management from generalised classification toward mechanism‐based precision medicine aimed at preserving endogenous insulin secretion and improving long‐term outcomes.

## Author Contributions

M.Z. wrote the paper and prepared the figures.

## Funding

This work was supported by China Medical University (Taichung, Taiwan), and by a Senior Jade Mountain Award (Ministry of Education, Taipei, Taiwan).

## Ethics Statement

The author has nothing to report.

## Consent

The author has nothing to report.

## Conflicts of Interest

The author declares no conflicts of interest.

## Data Availability

The author has nothing to report.
